# Family caregivers’ willingness to use online psychological therapy for individuals with mental illness in China: a cross-sectional study

**DOI:** 10.3389/fpubh.2026.1833997

**Published:** 2026-06-03

**Authors:** Qianqian Li, Lijun Liu, Bingling Gao, Huipeng Ren, Miao Pan, Pingfang Zhong, Shixing Li, Chenhong Zhang, Xiaoxi Zhang, Jun Yan, Jinmin Liao

**Affiliations:** 1 Peking University Sixth Hospital, Peking University Institute of Mental Health, NHC Key Laboratory of Mental Health (Peking University), National Clinical Research Center for Mental Disorders (Peking University Sixth Hospital), Beijing, China; 2 Department of Psychological Medicine, Zhongshan Hospital (Xiamen), Fudan University, Xiamen, China; 3 Department of Psychiatry, First Hospital of Hebei Medical University, Shijiazhuang, Hebei, China; 4 The Second Affiliated Hospital of Xinxiang Medical University, Xinxiang, Henan, China; 5 The Third People's Hospital of Lincang, Lincang, Yunnan, China; 6 Ji’ao Brain Hospital of Siping, Jilin, China; 7 Shaanxi Nuclear Industry 215 Hospital, Xianyang, Shaanxi, China; 8 Department of Mental Health, Changzhi Medical College, Changzhi, Shanxi, China

**Keywords:** China, digital mental health, family caregivers, online psychotherapy, technology acceptance

## Abstract

**Introduction:**

In China, family caregivers often serve as gatekeepers to mental health services, influencing whether patients initiate or continue treatment. Understanding their attitudes toward digital interventions is therefore critical for the successful implementation of online psychotherapy programs.

**Objectives:**

To exploratorily examine caregivers’ willingness to support online psychotherapy, preferred delivery formats, and perceived benefits and barriers.

**Methods:**

A structured questionnaire was administered to 291 family caregivers recruited in psychiatric settings across multiple Chinese cities. The instrument assessed caregiver demographics, technology access, patient characteristics, and attitudes toward online psychotherapy, including willingness (0–100 visual analog scale), treatment preferences, perceived benefits and barriers and help-seeking stigma. Responses were analyzed using descriptive statistics, multiple linear regression, and deductive content categorization.

**Results:**

Caregivers reported moderate-to-high willingness (mean = 70.2 ± 26.7). The most preferred delivery format was therapist-guided individual online counseling (37.8%). The key perceived benefit was time savings, while unstable internet connectivity emerged as a primary barrier. Caregivers also favored brief treatment courses (≤10 sessions) and considered modest session fees (≤55.4 USD) acceptable. Multiple linear regression identified small-city residence (*β* = 0.216, *p* = 0.007) and female gender (*β* = −0.127, *p* = 0.042) as independent predictors of willingness, with modest explained variance (R^2^ = 0.081).

**Conclusion:**

Family caregivers in China are active decision-makers in mental health service utilization, not passive supporters. Their openness to online therapy is shaped by practical needs and confidence in clinician-led models. Caregivers in resource-scarce smaller cities show greater willingness, likely reflecting unmet local need, whereas female caregivers—who bear disproportionate care responsibilities—express more cautious attitudes. The modest explained variance highlights other influencing factors, necessitating further exploration. Successful digital mental health interventions must be co-designed with families, embedded in trusted healthcare institutions, and tailored to diverse socioeconomic contexts.

## 1 Introduction

In 2019, approximately one in eight people globally lived with a mental health condition, yet 71% of those affected did not receive any form of mental health services—a gap that has persisted across high-, middle-, and low-income countries (1). The COVID-19 pandemic further exacerbated the global burden of mental disorders, with estimates showing a 28% increase in major depressive disorder and a 26% rise in anxiety disorders in 2020 alone (2). In China, mental disorders have become the leading cause of health loss among children and adolescents as of 2021 (3), adding systemic pressures on an already strained mental health infrastructure.

In response, digital mental health solutions—particularly online psychotherapy delivered via video or phone—have rapidly scaled up as feasible, accessible alternatives to in-person care (4). Evidence supports their clinical effectiveness, including symptoms reduction in adolescents OCD (5) and high caregiver satisfaction with video-based CBT (6). Attitudes toward telehealth are generally favorable, as seen in a study where 59% of parents of children with ADHD in Ireland reported satisfaction with telehealth (7). Within China, research also indicated a moderate acceptability of digital mental health services among stakeholders (8), underscoring the critical role of familial support in digital health engagement (9).

However, a critical implementation gap remains—particularly in collectivist contexts like China, where family caregivers function as de facto gatekeepers to mental health services. Deeply rooted norms of familial interdependence (10, 11), culturally ascribed caregiving responsibilities (12), and their central role in healthcare navigation (13) collectively position caregivers as central decision-makers regarding treatment initiation and continuity. This role is further amplified by the uneven distribution of services, which are concentrated in urban areas with limited availability in rural regions (14–16). This geographical maldistribution imposes concrete access barriers on rural families—long travel distances, high costs, and prolonged waiting times—that frequently delay or prevent professional psychiatric care (17). Consequently, family caregivers in underserved areas must assume clinical tasks such as medication monitoring, crisis management, and psychosocial support that would otherwise fall to trained professionals. They typically do so without formal training or respite, amplifying both objective workload and subjective psychological distress (18, 19). As a result, families bear additional burdens and become key decision-makers in adopting new models of care, including digital mental health solutions (20, 21).

Despite this, existing research, particularly in the Chinese context, has two key limitations. First, most studies conflate patient and caregiver perspectives, failing to isolate caregivers’ unique attitudes and decision-making processes. Second, there is a lack of quantitative analysis on caregivers’ willingness and its determinants, including their socioeconomic background and perceptions of online psychotherapy. To better understand the underlying mechanisms of technology adoption in this context, the Technology Acceptance Model (TAM) offers a valuable theoretical lens. TAM posits that an individual’s behavioral intention to use a technology is primarily influenced by their perceived usefulness (PU) and perceived ease of use (PEOU) of that technology (22). While not the sole framework, considering these core constructs can provide a structured approach to interpreting caregivers’ attitudes toward online psychotherapy.

Addressing this gap is vital for designing family-centered models of online psychotherapy that are not only technically feasible but also socially acceptable and equitably implemented. Therefore, this study primarily aims to quantitatively assess the mean level of Chinese family caregivers’ willingness to support online psychotherapy, providing a reliable descriptive estimate in this understudied population. Additionally, it exploratorily investigates the sociodemographic and clinical factors shaping this willingness. By filling this critical evidence gap, our research aims to inform implementation strategies that ensure digital mental health solutions are effectively integrated into routine psychiatric care in ways that truly meet families’ needs.

## 2 Methods

### 2.1 Participants

This study was a cross-sectional, multi-center survey that employs a structured questionnaire to assess the attitudes and willingness of family caregivers of patients with mental disorders in China to support engagement in online psychotherapy. It was conducted and reported in accordance with the Strengthening the Reporting of Observational Studies in Epidemiology (STROBE) guidelines for cross-sectional studies.

We recruited primary family caregivers from eight hospitals across eight provinces in China, including both psychiatric specialty hospitals and general hospitals with psychiatric departments. The hospitals were located in cities classified into three tiers by population size (large: >1,000,000; medium: 500,000–1,000,000; small: <500,000). The study included caregivers of both outpatients and inpatients. All patients’ diagnoses were confirmed by attending psychiatrists according to ICD-10 criteria.

Caregiver eligibility was confirmed through a two-step screening process. Research staff first identified individuals accompanying the patient, then administered two screening questions to verify their status as the primary family caregiver (responsible for daily care and medical management) and to confirm they were aged 18 or above and capable of independent questionnaire completion. Only those who met these criteria and provided written informed consent were included in the study. For analytical purposes, caregivers were subsequently grouped by their relationship to the patient into three categories: core guardians (parents, spouses), secondary relatives (children, siblings), and others (other relatives, friends).

Eligible participants then scanned a QR code to access the questionnaire on the Wenjuanxing platform and completed it on their mobile devices. Data collection occurred over a two-month period (February to March 2025). The study was approved by the Ethics Committee of Peking University Sixth Hospital (Approval Number: (2025) Rapid Review [1–9-1]).

The sample size was estimated to ensure sufficient precision for the primary descriptive aim of this exploratory study: to accurately characterize the mean level of caregiver willingness. This approach aligns with the study’s primary goal of providing a reliable descriptive estimate of willingness in this understudied population. As prior data on this specific population were lacking, we calculated the sample size based on the survey data of a patient sample from our team’s previous research (23). The group’s mean score for willingness to receive psychotherapy was 70, with a standard deviation (*σ*) of 28.56. Setting a 95% confidence level (Z = 1.96) and a margin of error of 5 points, we used the formula below to calculate a required sample size of 126. Accounting for potential loss to follow-up and invalid questionnaires, we increased the sample size by 20%, resulting in a total of 152 participants needed. It is important to note that this sample size was calculated to achieve precision in estimating the mean score, and not specifically powered for the subsequent multivariate (regression and path) analyses, which are exploratory in nature.

Initial sample size equation:


$$n={\left(\frac{Z\cdot \sigma }{E}\right)}^{2}$$

### 2.2 Survey design

The survey was developed based on the prior work of our own research group and comprised five core sections: (1) Caregiver sociodemographics: Age, gender, residence, employment status, income level, educational background, marital status, and relationship to the patient. (2) Internet-connected device ownership and usage patterns. (3) Patient clinical characteristics: Primary diagnosis, treatment status, satisfaction with psychiatric medication, and perceived overall symptom improvement. (4) Attitudes toward and preferences for psychotherapy: This section assessed caregivers’ willingness to support online psychotherapy, along with associated decision-making factors. Specifically, it included: (a) Family caregivers’ willingness scores (on a visual analog scale of 0–100, where 0 = “completely unwilling” and 100 = “very willing”) for having patients participate in online psychotherapy; (b) Family caregivers’ beliefs about the need for psychotherapy, their concerns regarding treatment, and their preference rankings among modalities; (c) The benefits and barriers family caregivers perceive for patients receiving online psychotherapy, as well as their previous experience and expectations regarding the cost and treatment duration of online psychotherapy. (5) The Self-Stigma of Seeking Help Scale (SSOSH) was administered to measure help-seeking stigma and its association with online psychotherapy willingness. The scale has been validated for cross-cultural validity in various countries, including China (24), with scores ranging from 10 to 50 (higher scores indicate greater stigma). In this study, the SSOSH demonstrated acceptable internal consistency (Cronbach’s *α* = 0.69). The structured questionnaire was developed by the research team based on a comprehensive literature review and clinical experience. To ensure the quality and validity of the survey instrument, the following steps were undertaken prior to formal data collection:

- Content Validity: The draft questionnaire was reviewed by three senior attending psychiatrists and two psychotherapists for relevance to research objectives, clarity of wording, and cultural appropriateness. Items were revised based on their expert feedback.
- Pilot Testing: A pilot test with 20 caregivers assessed comprehensibility, completion time, and logical flow. No substantive modifications were required following this test.

It should be noted that because the questionnaire comprises distinct modules measuring different constructs (e.g., demographics, patterns of use, attitudes) and most items are categorical or ranking in format, calculating an overall Cronbach’s α coefficient for the entire instrument is not methodologically appropriate.

### 2.3 Statistical analysis

Statistical analysis was conducted using SPSS 26.0 (IBM, New York, NY, United States) and R 4.5.2 (R Core Team, 2022) for univariate analyses and multiple linear regression modeling. Descriptive statistics were presented as means with standard deviations (SD) for normally distributed continuous variables and frequencies with percentages for categorical variables.

To identify factors associated with the willingness to support online psychotherapy (treated as a continuous outcome, 0–100 scale), initial univariate analyses were performed. Independent-samples t tests or Mann–Whitney U tests were used for binary predictors, one-way analysis of variance (ANOVA) or Kruskal–Wallis tests for unordered categorical predictors, and Pearson or Spearman correlation coefficients for continuous/ordinal predictors. All variables with a significance level of *p* < 0.05 in these univariate analyses were considered as candidate predictors.

Subsequently, an exploratory multiple linear regression model was constructed. Candidate predictors identified from the univariate analyses, along with other theoretically relevant variables (e.g., patient age, caregiver gender, city size, patient medication status, and patient’s perceived overall improvement), were entered into this model to identify independent factors associated with willingness. Model assumptions (linearity, independence, homoscedasticity, normality of residuals) were satisfied, and multicollinearity was assessed via variance inflation factors (all VIFs < 10).

## 3 Results

### 3.1 Demographic and clinical characteristics

We collected 291 valid questionnaires from family caregivers who completed the survey. The mean age was 39.9 years (SD = 11.2), with 56.7% female. Most were core guardians (61.2%), followed by secondary relatives (27.8%) and other support systems (10.3%). The majority resided in medium or small cities (74.6%; see Table 1).

**Table 1:** Descriptive characteristics of the family caregivers.

Variables	*N* = 291
Age (years), mean ± SD	39.9 ± 11.2
Gender, n (%)	
Male	112 (38.5%)
Female	165 (56.7%)
Prefer not to disclose	14 (4.8%)
Relationship with the patient, n (%)	
Core guardians (parents + spouses)	178 (61.2%)
Secondary relatives (children + siblings)	81 (27.8%)
Other support systems (relatives + friends)	30 (10.3%)
NA	2 (0.7%)
Residence, n (%)	
Large city	74 (25.4%)
Middle city	126 (43.3%)
Small city	91(31.3%)
Employment status, n (%)	
Full-time	160 (55.0%)
Part-time	32 (11.0%)
Unemployed	14 (4.8%)
Retired	23 (7.9%)
Others	62 (21.3%)
Annual income, n (%)	
Below 10,000 yuan	54 (18.6%)
10,000–50,000 yuan	81 (27.8%)
50,000–100,000 yuan	59 (20.3%)
100,000–200,000 yuan	33 (11.3%)
200,000–500,000 yuan	15 (5.2%)
Above 500,000 yuan	10 (3.4%)
Prefer not to say	39 (13.4%)
Marital status, n (%)	
Married	219 (75.3%)
Unmarried/divorced/widowed/Others	72 (24.7%)
Educational level, n (%)	
Primary school	38 (13.1%)
Junior high school	52 (17.9%)
Senior high school	37 (12.7%)
Higher Vocational Education	57 (19.6%)
Bachelor’s Degree	75 (25.8%)
Master’s Degree	21 (7.2%)
Doctoral Degree	11 (3.8%)
Usage of network devices (Multiple Selection), n (%)	
Smartphones	284 (97.6%)
Desktop Computers	84 (28.9%)
Laptop Computers	120 (41.2%)
Tablet Computers	102 (35.1%)
Game Consoles	24 (8.2%)
Others	2 (0.7%)
Internet usage frequency, n (%)	
Almost all the time	147 (50.5%)
Several times a day	95 (32.6%)
Approximately once a day	16 (5.5%)
Several times a week	14 (4.8%)
Once a week or less	19 (6.5%)
Total score of SSOSH, mean (SD)	24.0 ± 5.5

SSOSH, Self-Stigma of Seeking Help Scale. NA, not available.

Patients averaged 32.5 years (SD = 17.6), and 63.6% had anxiety/depression. Most were satisfied with current pharmacological treatment (79.5%) and reported improvement (88.0%). A minority (21.9%) had tried brief online psychotherapy (≤5 sessions), with moderate satisfaction regarding privacy (62.3%) and quality (65.6%; see Table 2).

**Table 2:** Patient characteristics: family perspective.

Variables	*N* = 291
Age (years), mean ± SD	32.5 ± 17.6
Current disease diagnosis, n (%)	
Anxiety	75 (25.8%)
Depression	110 (37.8%)
Obsessive-Compulsive Disorder	12 (4.1%)
Sleep Disorder	23 (7.9%)
Bipolar Disorder	16 (5.5%)
Trauma-Related Disorder	2 (0.7%)
Substance Dependence-Related Disorder	5 (1.7%)
Psychotic Disorder	35 (12.0%)
Other	13 (4.5%)
Course of disease (years) median (Q1-Q3)	1.3 (0.5–3.3)
Taking psychiatric medications, n (%)	210 (72.2%)
Satisfaction with medication, n (%)	
Very satisfied	60 (28.6%)
Relatively satisfied	107 (51.0%)
Neutral	38 (18.1%)
Relatively dissatisfied	5 (2.4%)
Very dissatisfied	0 (0%)
Degree of disease improvement, n (%)	
Very significant improvement	59 (20.3%)
Significant improvement	109 (37.5%)
Slight improvement	88 (30.2%)
No change	33 (11.3%)
Slight deterioration	2 (0.7%)
Significant and very significant deterioration	0 (0%)
Treatment approach, n (%)	
Outpatient treatment	156 (53.6%)
Inpatient treatment	98 (33.7%)
Day treatment	12 (4.1%)
Other	25 (8.6%)
Reasons for patients seeking psychotherapy	
Emotional issues such as anxiety and depression	236 (81.1%)
Physical-related problems such as eating disorders	147 (50.5%)
Behavioral issues such as impulsivity and self-harm	89 (30.6%)
Study/work pressure	106 (36.4%)
Interpersonal relationship troubles	91 (31.3%)
Effects caused by past trauma	60 (20.6%)
Substance dependence-related problems	34 (11.7%)
Psychotic symptoms	57 (19.6%)
Others	4 (1.4%)
What concerns do patients have about psychotherapy	
Worry that others will think they have problems	141 (48.5%)
Fear of being looked down on if others find out	106 (36.4%)
Lack of knowledge about mental health	133 (45.7%)
Belief that psychotherapy cannot help themselves	55 (18.9%)
Prefer to solve problems on their own	52 (17.9%)
Other	26 (8.9%)
Have used online psychotherapy, n (%)	61 (21.0%)
Number of times online psychotherapy has been used	
Five times or fewer	37 (60.7%)
More than six times	19 (31.1%)
Do not know	5 (8.2%)
The delivery methods of online psychotherapy	
Mainly via video or phone or both	46 (75.4%)
Text (messages)	11 (18.0%)
Others	4 (6.6%)
Satisfaction of patients (*n* = 61) with the privacy/security of previously attended online psychotherapy	61(21.9%)
Dissatisfied	7 (11.5%)
Neutral	13 (21.3%)
Satisfied	38 (62.3%)
Do not know	3 (4.9%)
Overall satisfaction of patients (*n* = 61) with previously attended online psychotherapy	61(21.9%)
Dissatisfied	4(6.6%)
Neutral	15 (24.6%)
Satisfied	40 (65.6%)
Do not know	2 (3.3%)

### 3.2 Willingness, preference, and perceived benefits and barriers

Family caregivers expressed moderate to high willingness to support the patient’s participation in online psychotherapy (mean = 70.2, SD = 26.7, on a 0–100 scale). When asked about preferred formats, the most selected option was therapist-guided online individual therapy (37.8%), followed by face-to-face individual therapy (25.8%). All other formats—including online group, self-guided, AI-based, VR, and face-to-face group therapy—were each chosen by less than 15% of respondents (see Table 3).

**Table 3:** Family caregivers’ willingness and preference for psychotherapy.

Variables	*N* = 291
Willingness of engage in online psychotherapy, mean (SD)	70.2 ± 26.7
Psychotherapy modalities ranked by preference/willingness	
Online individual therapy (with therapist)	110 (37.8%)
Face-to-face individual therapy	75 (25.8%)
Online group therapy (with therapist)	32 (11.0%)
AI-based psychotherapy	32 (11.0%)
Self-guided online therapy (e.g., apps/mini-programs)	21 (7.2%)
Virtual reality (VR) therapy	11 (3.8%)
Face-to-face group therapy	10 (3.4%)
Acceptable cost ranges for patients per session of online psychotherapy	
≤400 yuan / session	256 (88.0%)
401–600 yuan / session	17 (5.8%)
>600 yuan / session	18 (6.2%)
The expected course of online psychotherapy for patients’ participation	
1–5 sessions	131 (45.0%)
6–10 sessions	102 (35.1%)
11–15 sessions	41 (14.1%)
>15 sessions	17 (5.8%)

Most caregivers (88.0%) considered a per-session fee of ≤ 400 CNY (= 55.4 USD) acceptable and preferred brief treatment courses, with nearly half opting for 1–5 sessions and approximately 80% selecting ≤10 sessions in total (see Table 3).

**Figure 1:**
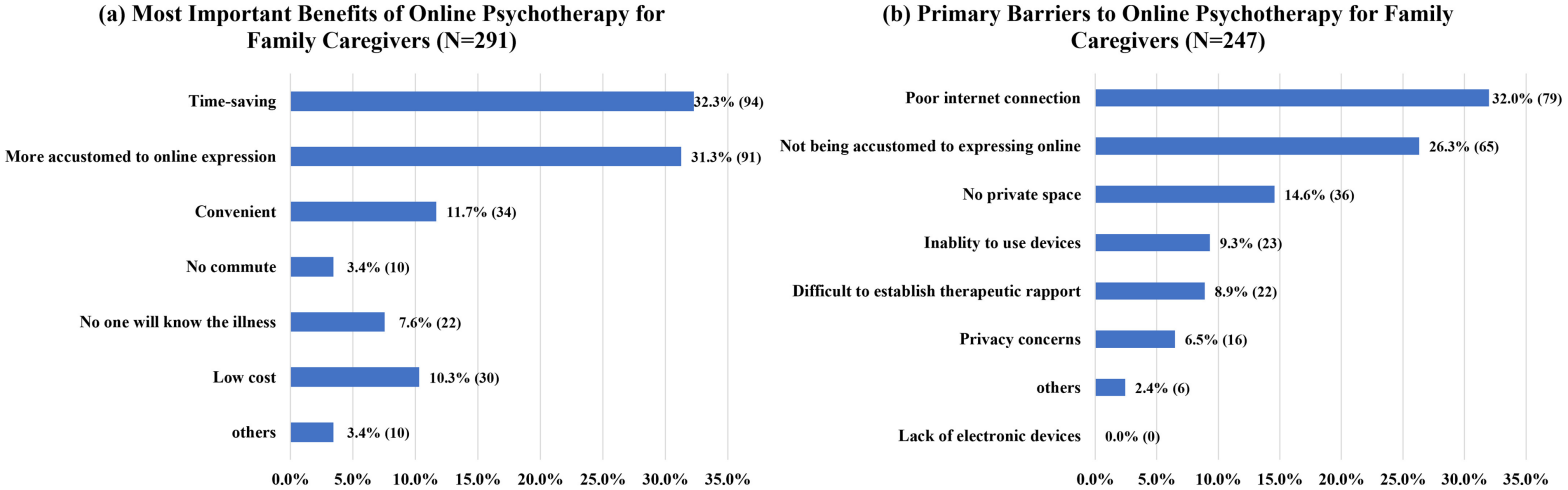
Perceived Benefits and Barriers to Online Psychotherapy for Family Caregivers. The bar chart displays the benefits or barriers of online psychological therapy on the y-axis, with percentages on the x-axis. Each bar represents the percentage and number of family caregivers who rated the corresponding benefit or barrier as the most important, with the number of respondents indicated in parentheses.

For family caregivers, the most commonly reported benefits of online psychotherapy were time savings (32.3%) and greater comfort in expressing emotions online (31.3%). In contrast, the primary barriers included unstable internet connections (e.g., disconnections, audio delays, signal instability; 32.0%) and discomfort with online communication (26.3%; see Figure 1). Among those citing unstable internet connections as a barrier (32.0%), 58.2% were residents of medium-sized cities. No statistically significant differences in sociodemographic or clinical characteristics were found between caregivers who perceived online expression as a benefit versus a barrier.

### 3.3 Factors associated with willingness to support online psychotherapy

#### 3.3.1 Univariate analysis

Univariate analyses identified several factors associated with caregivers’ willingness to support online psychotherapy. Patient age showed a significant positive correlation with willingness (Spearman’s rho = 0.149, *p* = 0.012). Caregiver residence (Kruskal–Wallis χ^2^ (2) = 12.56, *p* = 0.002), satisfaction with patient medication (χ^2^ (3) = 11.48, *p* = 0.009), and degree of patient improvement (χ^2^ (4) = 12.97, *p* = 0.011) were also significantly associated with willingness. Caregiver gender exhibited a marginal association (χ^2^ (2) = 5.98, *p* = 0.050). Other demographic and psychological variables did not reach statistical significance (see Supplementary Table S1).

#### 3.3.2 Multiple linear regression analysis

A multiple linear regression model was constructed to exploratorily identify independent predictors of willingness. The overall model was statistically significant (*F* (9, 274) = 2.69, *p* = 0.005) and explained 8.1% of the variance (R^2^ = 0.081), with an adjusted R^2^ of 0.051. Caregiver residence and gender emerged as significant independent predictors within this exploratory model. Specifically, caregivers residing in small cities reported higher willingness compared to those in large cities (B = 12.427, *β* = 0.216, *p* = 0.007). Female caregivers showed lower willingness than male caregivers (B = −6.874, *β* = −0.127, *p* = 0.042). Patient age, medication status, and improvement level were not significant in the multivariate model. Model diagnostics indicated no multicollinearity (all VIF < 2) and homoscedasticity was satisfied (Breusch–Pagan test, *p* = 0.069; see Table 4).

**Table 4:** Multiple linear regression analysis predicting family caregivers’ willingness to online psychotherapy (*N* = 284).

Variables	B	SE	β	t	*p*
(Intercept)	59.791	5.863		10.197	<0.001
Patient Age	0.148	0.092	0.097	1.604	0.110
Gender_Factor					
Female	−6.874	3.369	−0.127	−2.041	0.042*
Prefer not to say	−10.512	7.621	−0.085	−1.379	0.169
CitySize_Factor					
Medium City	4.183	4.077	0.077	1.026	0.306
Small City	12.427	4.546	0.216	2.733	0.007**
IsOnMedication_Factor					
On Medication	−1.051	3.975	−0.018	−0.264	0.792
OverallImprovement_Factor_Combined					
Very Significantly Improved	8.761	6.262	0.130	1.399	0.163
Significantly Improved	5.199	5.689	0.094	0.914	0.362
Slightly Improved	3.452	5.624	0.059	0.614	0.540

R^2^ = 0.081, Adjusted R^2^ = 0.051, F(9, 274) = 2.69, *p* = 0.005. *p < 0.05, **p < 0.01. Reference groups: Gender_Factor (Male), CitySize_Factor (Large City), IsOnMedication_Factor (Not on Medication), OverallImprovement_Factor_Combined (No Change or Worsened).

## 4 Discussion

This study examined Chinese family caregivers’ willingness to support patients’ participation in online psychotherapy, their format preferences, perceived benefits and barriers, and associated sociodemographic factors. Drawing on the Technology Acceptance Model (TAM) as an interpretive framework (22), we contextualized caregivers’ perceptions within the dual constructs of Perceived Usefulness (PU) and Perceived Ease of Use (PEOU) to understand how these cognitions shape behavioral intention in a collectivist cultural setting where families serve as primary gatekeepers for mental health treatment decisions.

### 4.1 Willingness and format preferences

Family caregivers expressed a moderately high willingness to support online psychotherapy (mean = 70.2/100), suggesting considerable openness despite well-documented cultural barriers to psychiatric help-seeking in Chinese society. This finding aligns with TAM, as it reflects caregivers’ initial positive perceptions of online psychotherapy. It also resonates with a South Korean study on dementia caregivers, where PU and PEOU of non-face-to-face services significantly predicted caregivers’ intention to use them (25). In China, families often make mental health decisions, so this high willingness is a key factor for promoting digital mental health services.

The strong preference for therapist-guided online individual therapy (37.8%)—over unguided, AI-based, group, or VR formats—indicates that caregivers value professional involvement and interpersonal accountability while seeking the logistical advantages of digital delivery. This is consistent with findings from Teles et al., who reported that digitally skilled dementia caregivers preferred online interventions that allowed interaction with professionals and featured friendly interfaces, even when they were otherwise comfortable with technology (26). Schwedler et al. similarly observed that family caregivers’ perceived benefits of digital health systems are shifting from purely functional support toward interactive, communicative dimensions, underscoring the importance of maintaining human therapeutic involvement (27). The additional preference for brief treatment courses (≤10 sessions) and modest per-session fees (≤55.4 USD) reflects pragmatic constraints inherent to the caregiving role—time scarcity, financial burden, and the need for minimal disruption to existing routines. Taken together, these preferences indicate that therapist-guided online psychotherapy functions not merely as a digital substitute for in-person care, but as a culturally and practically adaptive model that balances professional quality with accessibility.

### 4.2 Perceived benefits and barriers through a TAM lens

Within the TAM framework, PU refers to the degree to which a user believes a technology will enhance performance, while PEOU captures the expected effort required (22). Linking caregivers’ views to these two constructs helps identify key factors influencing their willingness. The most common benefits reported were time savings (32.3%) and greater comfort in online emotional expression (31.3%). These align with PU, as they reduce caregiving burden and promote treatment engagement. These findings parallel those from a study of family caregivers in Japan, where PU of assistive technologies was driven by tangible caregiver and workplace benefits rather than abstract technological advantages (27). A qualitative study by Turk et al. also found that informal carers recognized the potential value of personalized web-based resources, emphasizing convenience and accessibility as key drivers of PU (28).

Conversely, the primary barriers—unstable internet connections (32.0%) and discomfort with online communication (26.3%)—correspond to the PEOU construct. Notably, 58.2% of those with connectivity issues lived in medium-sized cities, indicating an infrastructure gap in non-metropolitan areas. This gap directly reduces PEOU, as poor internet makes online psychotherapy hard to use. This parallels findings from a study of mobile health acceptance among cancer patients and caregivers in a lower-middle-income country, where urban–rural divides in network quality emerged as a critical barrier to PEOU (29). In China, Tang et al. showed that PEOU of online medical websites affected behavioral intention through PU and trust—poor infrastructure not only reduces ease of use but also trust in online services (30). Similarly, Tao et al. found that Chinese caregivers, despite performing well, worried more about technology complexity, possibly due to less prior experience (31). Thus, addressing PEOU barriers requires two key steps: improving network infrastructure and providing digital literacy support.

Notably, sociodemographic and clinical characteristics did not differ between caregivers who saw online expression as a benefit or a barrier. This supports TAM, which emphasizes that individual perceptions (e.g., digital experience, personality) rather than structural factors drive behavioral intention.

### 4.3 Independent predictors of willingness

The multiple regression model (*F* (9, 274) = 2.69, *p* = 0.005, R^2^ = 0.081) identified two independent predictors of willingness after controlling for other significant univariate factors.

Small-city residence (B = 12.43, *β* = 0.216, *p* = 0.007) was the strongest predictor. Caregivers in smaller cities reported substantially higher willingness than those in large cities, likely reflecting a pragmatic response to limited local mental health resources. Consistent with TAM, PU increases when technology meets unmet needs (22)—online psychotherapy addresses small-city caregivers’ urgent need for accessible mental health services. Chinese studies also show that residents in resource-constrained areas are more likely to accept internet health services due to necessity (30, 32).

Female gender (B = −6.87, *β* = −0.127, *p* = 0.042) was associated with lower willingness. This gender difference warrants careful interpretation. Xiong et al. found that female dementia caregivers knew more about care technology but were less willing to invest financially (33). In China, two reasons may explain this: first, women bear most caregiving responsibilities and have higher expectations for therapeutic relationships, viewing online modalities as less effective; second, their heavy care burden makes them hesitant to add new treatment tasks. A study examining technology use among Chinese immigrant caregivers also identified potential barriers including limited free time and caregiving burdens—factors that may disproportionately affect female caregivers (34). Future qualitative research is needed to disentangle these mechanisms.

Variables significant in univariate analyses (patient age, medication satisfaction, perceived improvement) were not significant in the multivariate model. This aligns with TAM: external and demographic factors influence willingness indirectly through PU and PEOU, not directly (35). Aksin et al. similarly found that caregiver burden and technology affinity, not disease stage, predicted engagement intention in dementia caregivers (36).

### 4.4 Theoretical implications: TAM in mental health caregiving

The relatively modest explained variance (adjusted R^2^ = 0.051) merits both acknowledgment and theoretical reflection. Several considerations apply.

First, the TAM framework was originally developed for information technology adoption in organizational settings (22). Its core constructs (PU, PEOU) capture cognitive evaluations of technology attributes. However, mental health treatment decisions are embedded in emotional, relational, and cultural contexts that extend beyond cognitive technology appraisal. An umbrella review of digital intervention acceptance among dementia caregivers identified that acceptance factors and barriers span technological, personal, social, and organizational dimensions—many of which are not captured by traditional TAM constructs (37). In our study, the TAM-derived PU and PEOU constructs provided a useful organizing vocabulary for understanding caregivers’ perceived benefits and barriers, but the regression results suggest that substantial variance in willingness remains attributable to unmeasured factors.

Second, caregivers’ willingness is a proxy decision, involving not only their own cognitions but also patient readiness, therapeutic relationships, and family dynamics. TAM can be complemented by the Health Belief Model, which includes threat perception, self-efficacy, and other factors relevant to health decisions. Hiraga et al. proposed that the TAM requires context-specific modification when applied to caregiving, as PU operates through distinct caregiver-specific benefit pathways rather than the generic performance expectancy assumed in the original model (35).

Third, the measurement approach for PU and PEOU in this study—forced ranking of the given benefits and barriers—differs from conventional Likert-scale assessments used in TAM research. This ipsative format, while ecologically valid in reflecting real-world prioritization, compresses inter-individual variance and may attenuate statistical associations. Future studies should employ independent rating scales for each benefit and barrier item to better capture the full range of TAM constructs.

### 4.5 Clinical implications

These findings translate into actionable recommendations for digital mental health service design and implementation in China:

1. Prioritize small-city populations. The convergence of highest unmet need and greatest willingness in smaller cities suggests these regions should be primary targets for platform deployment, clinician recruitment, and awareness campaigns—while simultaneously investing in network infrastructure to address connectivity barriers.
2. Maintain therapist involvement. The strong preference for guided formats indicates that fully automated or AI-only solutions are premature for this population. Hybrid models combining digital convenience with professional therapeutic relationships are most likely to achieve sustained engagement (26, 27).
3. Address female caregivers’ specific concerns. Service design should explicitly attend to quality assurance, therapeutic relationship building, and integration with existing caregiving demands to reduce potential reluctance among female caregivers (33).
4. Enhance digital onboarding. Given that discomfort with online communication was a leading barrier, platforms should offer simplified interfaces, guided tutorials, and offline onboarding support—particularly for caregivers with limited digital experience (31).
5. Design for pragmatic constraints. Services should adopt brief courses (≤10 sessions) and affordable fees (≤55.4 USD) to minimize time and financial burden on caregivers.

### 4.6 Limitations

Several limitations should be acknowledged. First, the cross-sectional design precludes causal inference; associations identified may reflect unmeasured confounders or reverse causality. Second, the sample was recruited from psychiatric and general hospital outpatient settings at a limited number of sites, potentially excluding community-dwelling caregivers or those in rural areas with the highest telehealth need. This recruitment method, utilizing QR code-based online surveys, may have preferentially included caregivers with higher digital literacy, greater engagement in care, and more favorable attitudes toward online interventions, potentially leading to an overestimation of willingness to support online psychotherapy. Third, PU and PEOU were measured through forced-ranking of top-three items rather than independent Likert scales, which constrains variance and limits direct comparability with standard TAM operationalizations. Fourth, the relatively low explained variance (R^2^ = 0.081) indicates that important predictors remain unidentified; future studies should examine trust in specific platforms, prior therapeutic alliance experiences, patient willingness, and family communication patterns. Fifth, while patient age, medication satisfaction, and perceived improvement reached significance in univariate analyses, they did not survive multivariate adjustment, underscoring their hypothesis-generating rather than confirmatory nature. Finally, willingness was assessed with a single 0–100 item; multi-item validated instruments measuring behavioral intention would improve measurement precision in future research.

## Data Availability

The raw data supporting the conclusions of this article will be made available by the authors upon reasonable request directed to the corresponding author.
