# Correction: Family caregivers' willingness to use online psychological therapy for individuals with mental illness in China: a cross-sectional study

**DOI:** 10.3389/fpubh.2026.1920135

**Published:** 2026-07-13

**Authors:** Qianqian Li, Lijun Liu, Bingling Gao, Huipeng Ren, Miao Pan, Pingfang Zhong, Shixing Li, Chenhong Zhang, Xiaoxi Zhang, Jun Yan, Jinmin Liao

**Affiliations:** 1 Peking University Sixth Hospital, Peking University Institute of Mental Health, NHC Key Laboratory of Mental Health (Peking University), National Clinical Research Center for Mental Disorders (Peking University Sixth Hospital), Beijing, China; 2 Department of Psychological Medicine, Zhongshan Hospital (Xiamen), Fudan University, Xiamen, China; 3 Department of Psychiatry, First Hospital of Hebei Medical University, Shijiazhuang, Hebei, China; 4 The Second Affiliated Hospital of Xinxiang Medical University, Xinxiang, Henan, China; 5 The Third People's Hospital of Lincang, Lincang, Yunnan, China; 6 Ji'ao Brain Hospital of Siping, Jilin, China; 7 Shaanxi Nuclear Industry 215 Hospital, Xianyang, Shaanxi, China; 8 Department of Mental Health, Changzhi Medical College, Changzhi, Shanxi, China

**Keywords:** China, digital mental health, family caregivers, online psychotherapy, technology acceptance

Affiliation 1 “Peking University Sixth Hospital, Peking University Institute of Mental Health, NHC Key Laboratory of Mental Health (Peking University), National Clinical Research Center for Mental Disorders (Peking University Sixth Hospital), Beijing, China” and affiliation 2 “Department of Psychological Medicine, Zhongshan Hospital (Xiamen), Fudan University, Xiamen, China” were erroneously given as 1. “NHC Key Laboratory of Mental Health, National Clinical Research Center for Mental Disorders, Peking University Institute of Mental Health, Peking University Sixth Hospital, Beijing, China” and 2. “Department of Psychological Medicine, Zhongshan Hospital, Fudan University, Xiamen, China”.

The original version of this article has been updated.

