# Supplementary material for: Family caregivers’ willingness to use online psychological therapy for individuals with mental illness in China: a cross-sectional study
Source: Front Public Health. 2026 Jun 3;14:1833997. doi: 10.3389/fpubh.2026.1833997 (PMC13272136; doi:10.3389/fpubh.2026.1833997)
Supplement: Supplementary file 1 [file Table_1.docx]

Supplementary Material

# Supplementary Tables

**
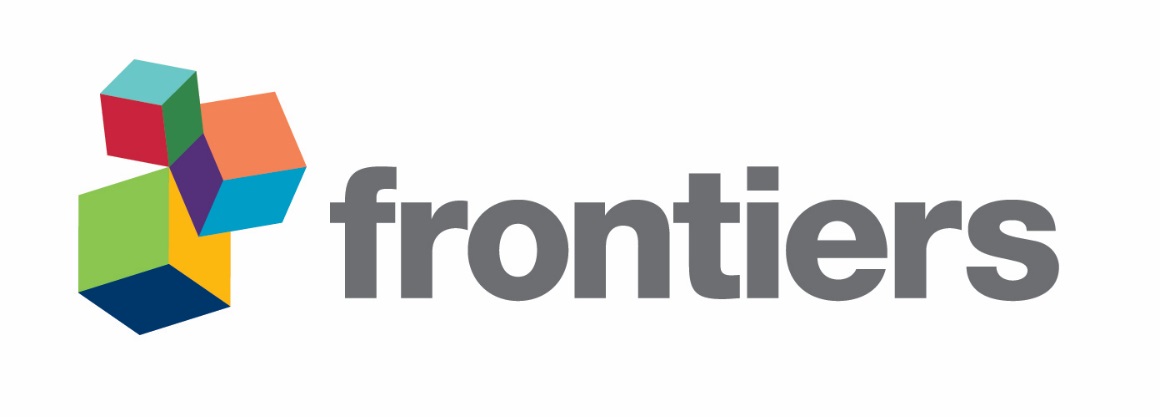
**

**Supplementary** **Table S1.** **Descriptive Statistics and Univariate Analysis of Factors Associated with Caregivers' Willingness to Use Online Psychotherapy​**

| Variable | n (%) or Mean (SD) | Univariate Analysis (Test Statistic, df, *p*-value) |
| --- | --- | --- |
| ​​Willingness of engage in online psychotherapy | 70.2 (26.7) |  |
| ​​**Caregiver Demographics​​** |  |  |
| Caregiver Age (years) | 39.9 (11.2) | Spearman's rho = -0.046, *p* = 0.441 |
| Patient Age (years) | 32.5 (17.6) | Spearman's rho = 0.149, *​​p* = 0.012​​* |
| Caregiver Gender |  | Kruskal-Wallis $\chi^{2}$ (2) = 5.98, *p* = 0.050 |
| Male | 112 (38.5%) |  |
| Female | 165 (56.7%) |  |
| Prefer not to disclose | 14 (4.8%) |  |
| Residence |  | Kruskal-Wallis $\chi^{2}$ (2) = 12.56, *​​p* = 0.002* |
| Large City | 74 (25.4%) |  |
| Middle City | 126 (43.3%) |  |
| Small City | 91 (31.3%) |  |
| Education Level |  | Kruskal-Wallis $\chi^{2}$ (6) = 8.36, *p* = 0.213 |
| Primary/Below | 38 (13.1%) |  |
| Junior High School | 52 (17.9%) |  |
| Senior High school | 37 (12.7%) |  |
| Higher Vocational | 57 (19.6%) |  |
| Bachelor's Degree | 75 (25.8%) |  |
| Master's Degree | 21 (7.2%) |  |
| Doctoral Degree | 11 (3.8%) |  |
| Marital Status |  | Kruskal-Wallis $\chi^{2}$ (4) = 6.82, *p* = 0.146 |
| Single | 59 (20.3%) |  |
| Married | 219 (75.3%) |  |
| Divorced | 6 (2.1%) |  |
| Widowed | 5 (1.7%) |  |
| Other | 2 (0.7%) |  |
| Employment Status |  | Kruskal-Wallis $\chi^{2}$ (4) = 6.46, *p* = 0.167 |
| Employed (Full-time) | 160 (55.0%) |  |
| Employed (Part-time) | 32 (11.0%) |  |
| Unemployed | 14 (4.8%) |  |
| Retired | 23 (7.9%) |  |
| Others | 62 (21.3%) |  |
| ​​**Clinical Factors​​** |  |  |
| Satisfaction with Medication |  | Kruskal-Wallis $\chi^{2}$ (3) = 11.48, *​​p* = 0.009* |
| Very Satisfied | 60 (28.6%) | ​​ |
| Relatively Satisfied | 107 (51.0%) |  |
| Neutral | 38 (18.1%) |  |
| Relatively Dissatisfied | 5 (2.4%) |  |
| Very Dissatisfied | 0 (0.0%) |  |
| Degree of disease improvement |  | Kruskal-Wallis $\chi^{2}$ (4) = 12.97, *​​p* = 0.011* |
| Very significant improvement | 59 (20.3%) |  |
| Significant improvement | 109 (37.5%) |  |
| Slight improvement | 88 (30.2%) |  |
| No change | 33 (11.3%) |  |
| Slight deterioration | 2 (0.7%) |  |
| ​​**Psychological Factors​​** |  |  |
| SSOSH Total Score | 24.0 (5.5) | Spearman's rho = -0.087, *p* = 0.139 |

*Note:* * *p* < 0.05, ** *p* < 0.01. n represents the number of participants for categorical variables, and Mean (SD) represents the mean and standard deviation for continuous variables. Univariate analyses were conducted to examine the association between each independent variable and family caregivers' willingness to engage in online psychotherapy. Spearman's rank correlation was used for continuous variables, and Kruskal-Wallis H test was used for categorical variables.
